# Selective Hyaluronan–CD44 Signaling Promotes miRNA-21 Expression and Interacts with Vitamin D Function during Cutaneous Squamous Cell Carcinomas Progression Following UV Irradiation

**DOI:** 10.3389/fimmu.2015.00224

**Published:** 2015-05-13

**Authors:** Lilly Y. W. Bourguignon, Daniel Bikle

**Affiliations:** ^1^Endocrine Unit (111N2), Department of Medicine, San Francisco Veterans Affairs Medical Center, University of California at San Francisco, San Francisco, CA, USA

**Keywords:** hyaluronan, CD44, RhoGTPase, vitamin D, UVR, miR21, vitamin D, skin cancer

## Abstract

Hyaluronan (HA), the major extracellular matrix component, is often anchored to CD44, a family of structurally/functionally important cell surface receptors. Recent results indicate that UV irradiation (UVR)-induced cutaneous squamous cell carcinomas (SCC) overexpress a variety of CD44 variant isoforms (CD44v), with different CD44v isoforms appear to confer malignant SCC properties. UVR also stimulates HA degradation in epidermal keratinocytes. Both large HA polymers and their UVR-induced catabolic products (small HA) selectively activate CD44-mediated cellular signaling in normal keratinocytes and SCC cells, with all of the downstream processes being mediated by RhoGTPases (e.g., Rac1 and Rho). Importantly, we found that the hormonally active form of vitamin D 1,25(OH)_2_D_3_ not only prevents the UVR-induced small HA activation of abnormal keratinocyte behavior and SCC progression, but also enhances large HA stimulation of normal keratinocyte activities and epidermal function(s). The aim of this hypothesis and theory article is to question whether matrix HA and its UVR-induced catabolic products (e.g., large and small HA) can selectively activate CD44-mediated cellular signaling such as GTPase (Rac and RhA) activation. We suggested that large HA–CD44 interaction promotes Rac-signaling and normal keratinocyte differentiation (lipid synthesis), DNA repair, and keratinocyte survival function. Conversely, small HA–CD44 interaction stimulates RhoA activation, NFκB/Stat-3 signaling, and miR-21 production, resulting in inflammation and proliferation as well as SCC progression. We also question whether vitamin D treatment displays any effect on small HA–CD44v-mediated RhoA signaling, inflammation, and SCC progression, as well as large HA–CD44-mediated differentiation, DNA repair, keratinocyte survival, and normal keratinocyte function. In addition, we discussed that the topical application of signaling perturbation agents (e.g., Y27623, a ROK inhibitor) may be used to treat certain skin diseases displaying upregulation of keratinocyte proliferation such as psoriasis and actinic keratoses in order to correct the imbalance between Rac and RhoA signaling during various UV irradiation-induced skin diseases in patients. Finally, we proposed that matrix HA/CD44-signaling strategies and matrix HA (HA_S_ vs. HA_L_ or HA_S_ → HA_L_)-based therapeutic approaches (together with vitamin D) may be used for the treatment of patients suffering a number of UV irradiation-induced skin diseases (e.g., inflammation, skin cancer, and chronic non-healing wounds).

## Introduction

The incidence of skin cancer has been increasing rapidly over recent decades. UVB radiation (UVR)-induced squamous cell carcinomas (SCC) is the second most common cancer among Caucasians in the United States, contributing substantially to morbidity among elderly people. Recent studies report that the age-adjusted incidence of SCC has grown by 50–200% over the past 10–30 years. Invasive SCC has the potential to recur locally, tending to invade deeply into subcutaneous structures, fascia, and muscle; and to metastasize, most commonly to regional lymph nodes (1, 2). Several lines of evidence indicate that matrix hyaluronan (HA) plays an important role in regulating inflammation, proliferation, and migration/invasion in the progression of a variety of tumors (3–7). Because little is known about the molecular basis underlying HA effects on influencing skin cancer development, there is currently a need to investigate some key aspects of HA signaling in regulating UVR-induced human SCC progression.

Normal keratinocytes and healthy skin tissues express predominantly one large species of the transmembrane glycoprotein, CD44 (Epican), required for many keratinocyte functions (3, 4). However, UVR-induced cutaneous SCC cells and tissues overexpress a variety of variant isoforms of CD44 (3–9). Different CD44 variant (CD44v) isoforms appear to confer the malignant properties of increased tumor cell growth and cancer progression (5–10). CD44v isoforms bind a number of extracellular matrix (ECM) ligands (e.g., HA), and are known to participate in a variety of both normal keratinocyte and SCC functions (3–10). Our recent results indicate that UVB stimulates HA degradation in normal keratinocytes. Both large HA polymers and their smaller catabolic products selectively activate CD44 isoform-mediated cellular signaling that regulates inflammation, anti-apoptosis, and tumor cell growth, as well as differentiation, DNA repair, and keratinocyte survival function.

### Skin cancer

Recent studies report that the age-adjusted incidence of skin cancer has grown by 50–200% over the past 10–30 years. Exposure to (UVR has well-recognized clinical effects on the skin, including sunburn (inflammation) and keratinocyte transformation, leading to neoplasia and SCC progression. In particular, skin type determines sensitivity to the acute and chronic effects of UVR on the skin. Today, highly aggressive variants of SCC are frequently seen in organ-transplant recipients, as well as in patients who are on immunosuppressive medications or have immunocompromised status for other reasons (1, 2). Human papillomavirus infection has also been associated with some types of cutaneous SCC. Invasive SCC has the potential to recur locally, tending to invade deeply into subcutaneous structures, fascia, and muscle; and to metastasize, primarily to the regional lymph nodes (1, 2). Because little is known about the molecular basis underlying the progression to the invasive phenotype, it is very difficult to predict individual tumor aggressiveness and design effective treatment plans. Thus, there is currently a real need to clarify aspects of tumor biology underlying the clinical behavior of SCC. It is well-known that the tumor-specific phenotype (characteristics such as inflammation, anti-apoptosis, and tumor cell proliferation) is linked to oncogenic signaling. Dissection of the transmembrane pathways controlling cellular signaling and tumor functions should significantly aid in understanding the intracellular events underlying SCC progression.

### Hyaluronan and CD44 in normal keratinocytes and squamous cell carcinomas

Hyaluronan, the major glycosaminoglycan of ECM component, serves not only as a primary constituent of connective tissue extracellular matrices but also as a bio-regulatory molecule (1, 2). Many studies indicate that HA is also abundant in stratified squamous epithelia, including mammalian epidermis, and that it influences epidermal functions such as skin integrity (10, 11). However, the mechanisms by which HA stimulates keratinocyte functions and regulates tissue integrity are not well understood. HA is synthesized by several HA synthases (12), and its size further modified by hyaluronidases (13). Generally, small size-HA (1 × 10^5^–1 × 10^4^ Da) induces the expression of proinflammatory cytokine/chemokine and proliferative genes as well as cell proliferation and migration; whereas large size-HA (>1 × 10^6^ Da) promotes transcriptional activation and differentiation (14–20). UVR-induced changes in HA production and degradation/fragmentation have also been reported (19, 20). Specifically, while large HA appears to predominate in normal mouse skin, small HA becomes prevalent in tumor tissues (19) and UVR-induced keratinocytes (20). All of these observations are consistent with our hypothesis that both HA production and HA size modifications underline UVR-induced changes associated with onset of keratinocyte transformation.

Both large and small HAs are capable of binding to CD44 (21), which is an ubiquitous, abundant, and functionally important receptor expressed on the surface of many cells, including normal and transformed keratinocytes (23). CD44 is encoded by a single gene which contains 19 exons (22). The most common form, CD44s (CD44 standard form), contains exons 1–5 (N-terminal 150 a.a.), exons 15 and 16 (membrane proximal 85 a.a.), exon 17 (transmembrane domain), and a portion of exons 17 and 19 (cytoplasmic tail, 70 a.a.). Out of the 19 exons, 12 exons can be alternatively spliced. Most often, the alternative splicing occurs between exons 5 and 15 leading to an insertion in tandem of one or more variant exons (exon 6–exon 14; v1–v10) within the membrane proximal region of the extracellular domain (31). For example, in keratinocytes, additional exons v3–v10 are inserted into the CD44s transcripts, and this isoform has been designated as CD44v3-10 (23). Various skin cancer cells and tissues express different CD44 variant (CD44v) isoforms (e.g., CD44v3 and CD44v10) in addition to CD44s and CD44v3-10 (23). These CD44 isoforms have the same amino acid sequences at the two ends of the molecule, but differ in the middle region (v3–v10) within the CD44 membrane proximal domain located at the external side of the membrane (23). Several lines of evidence clearly indicate that CD44 selects unique downstream effectors and coordinates certain downstream, intracellular signaling pathways that influence multiple cellular functions (23).

## Hypothesis

In this article, we described the hypothesis that HA and its UVR-induced catabolic products (e.g., large and small HA) selectively activate CD44-mediated Rac and RhoA signaling. Specifically, large HA–CD44 interaction promotes Rac/PKNγ and p38MAPK-dependent normal keratinocyte differentiation (lipid synthesis), DNA repair, and keratinocyte survival function. Conversely, small HA–CD44v isoform interaction stimulates RhoA/ROK-dependent NFκB/Stat-3 signaling and miR-21 production, resulting in inflammation and proliferation as well as SCC progression. Vitamin D treatment inhibits small HA–CD44v-mediated RhoA/ROK signaling, inflammation, and SCC progression, and it also enhances large HA–CD44-mediated differentiation, DNA repair, keratinocyte survival, and normal keratinocyte function. The results of this study will definitely provide a better understanding of the cellular and molecular mechanisms involved in normal keratinocyte-mediated epidermal function and UVR-induced human SCC progression, as well as vitamin D effects on reducing UVR-induced keratinocyte transformation and skin cancer.

## Experimental Evidence

### Detection of CD44v isoform expression in normal keratinocytes, SCC cells, and skin SCC tissues

**Figure 1:**
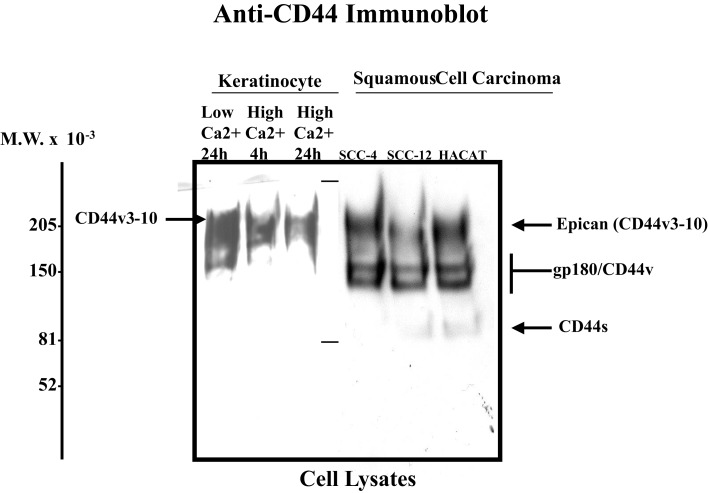
**Detection of CD44 isoform expression in normal keratinocytes and SCC cell lines using anti-CD44-mediated immunoblotting techniques [SCC-4, SCC-12 cell lines, and HACAT (a transformed cell line) Epican is designated as a CD44v3-10 form; anti-CD44-mediated immunoblot was used to detect CD44 signal as indicated]**.

**Figure 2:**
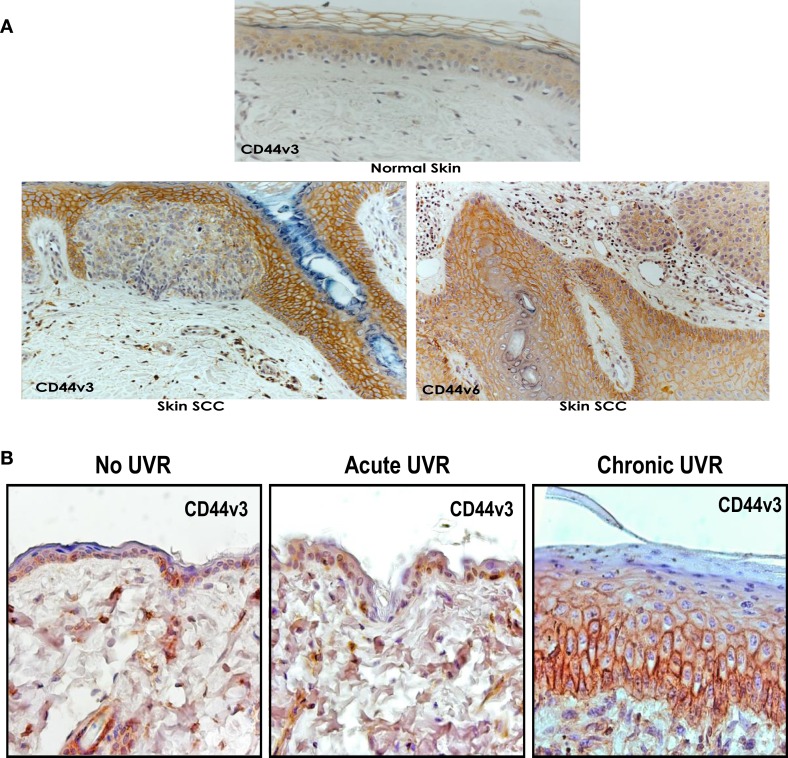
**(A)** Immunoperoxidase staining of CD44v3 and CD44v6 isoforms in normal and SCC tissues of human skin; and **(B)** immunoperoxidase staining of CD44v3 isoform in mouse skin following acute or chronic UVR exposure.

Invasive SCC induced by UVR has the potential to recur locally, tending to invade deeply into subcutaneous structures, fascia, and muscle, and to metastasize, preferably to the regional lymph nodes (1, 2). At the present time, very limited information is available regarding the factors responsible for the onset and progression of skin SCC. One promising candidate in this regard is the CD44 molecule. Our data using anti-CD44 antibody (recognizing the common domain of all CD44 isoforms) confirm that undifferentiated keratinocytes express various CD44 isoforms in abundance (Figure 1-low calcium-treated sample). However, following high Ca^2+^-induced keratinocyte differentiation, the lower molecular weight forms cease to be expressed leaving Epican as the dominant species (Figure 1). CD44v isoforms are overexpressed in cutaneous SCC cell lines as compared to normal keratinocytes (Figure 1). Furthermore, immunohistochemical staining using anti-CD44v3 antibody and anti-CD44v6 antibody confirms that both CD44v3 and CD44v6 isoforms are overexpressed in human cutaneous SCC tissue (Figure 2). Only a very small amount of CD44v isoforms is detected in normal skin tissue (Figure 2). CD44v3 overexpression can also be detected in mouse skin following chronic (to a lesser extent acute) UVB exposure (Figure 2). These findings clearly indicate a strong correlation between CD44v isoform expression and UVB-induced skin SCC progression.

### UVB-induced hyaluronan fragmentation and abnormal keratinocyte functions

**Figure 3:**
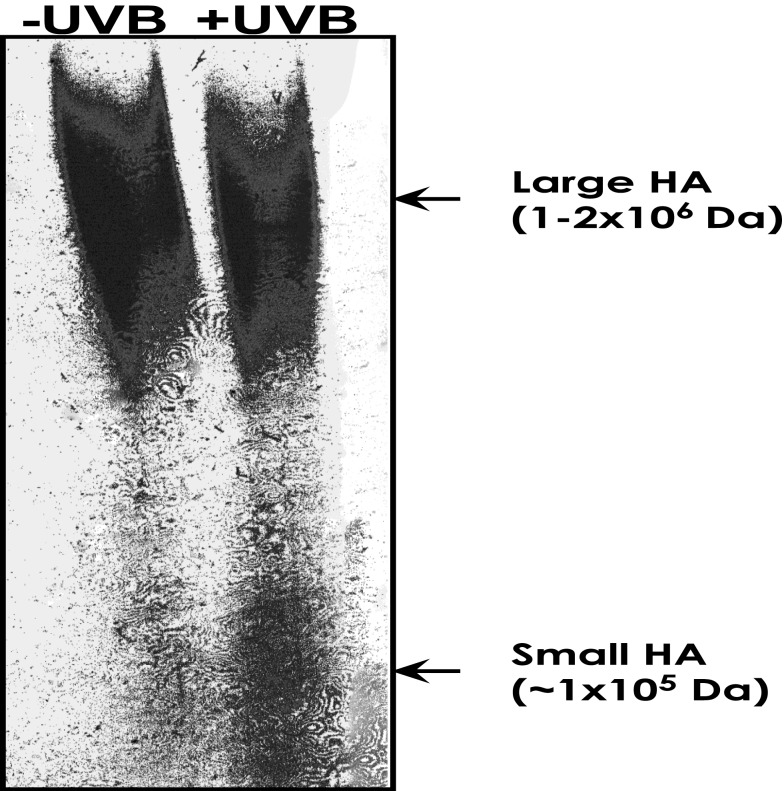
**Measurement of HA-size distribution in untreated mouse epidermis vs. acute UVB-treated mouse epidermis**. HA from untreated mouse epidermis vs. acute UVB-treated mouse epidermis was isolated and purified using gel filtration column chromatography, and then analyzed by an enzyme-linked binding protein assay, that uses microwells coated with a highly specific HA binding protein (HABP) to capture HA, and an enzyme-conjugated HABP using a commercially available enzyme-linked immunosorbent assay (ELISA)-type test kit. HA was also be analyzed by 0.5% agarose gel followed by Alcian blue and silver staining.

Hyaluronan belongs to one of the major glycosaminoglycan polysaccharide families and plays an important role in the formation/remodeling of ECM, specifically in maintaining the integrity of skin tissues (17, 18). Our data show that HA in normal epidermis (without UVB treatment) exists as a large polymer of approximately 1–2 × 10^6^ Da molecular weight (“large HA”) (Figure 3). These large HA polymers are degraded into smaller HA units (1 × 10^5^–1 × 10^3^ Da; “small HA”) following UVB treatment (Figure 3).

Both large and small HAs are capable of binding to CD44 – a ubiquitous, abundant, and functionally important receptor expressed on the surface of many cells including normal keratinocytes and SCC (Figure 2). Generally, small HA–CD44 interaction activates pro-inflammatory cytokine/chemokine gene expression/production, proliferation, and migration, whereas large HA–CD44 binding promotes certain transcriptional activation and differentiation (19, 20). HA–CD44 interaction induces intracellular RhoGTPase (RhoA and Rac) signaling cascades that regulate cytoskeletal reorganization and cell migration (18). Whether HA/CD44 and RhoGTPase signaling actually cause keratinocyte functions and SCC activation is not presently known, and is addressed below.

### HA/CD44-mediated RhoA and Rac1 signaling in cytoskeleton activation and keratinocyte and SCC functions

Members of the Rho subclass of the Ras superfamily [small molecular weight GTPases (e.g., RhoA, Rac1, and Cdc42)] act as molecular switches that alternate between GTP- and GDP-bound states. The “activated” GTP-bound enzymes preferentially interact with downstream effector molecules that modulate other effector activities (24). For example, activation of RhoA and Rac1 signaling has been shown to regulate cytoskeleton-associated functions in normal and transformed keratinocytes (SCC cells) (19, 20).

#### RhoA-Activated Rho-Kinase Signaling Events

Previous work indicated that HA (mixed sizes) promotes the interaction between CD44 and several Rho-specific guanine nucleotide exchange factors [e.g., p115RhoGEF (25) and LARG (26)], thereby up-regulating RhoA (a member of the Rho subclass of the Ras superfamily), leading to altered cytoskeleton-mediated cell functions (25, 26). For example, activation of RhoA signaling has been shown to be involved in cytoskeleton-associated functions. Several enzymes have been identified as possible downstream targets for RhoGTPases (e.g., RhoA) in regulating cytoskeleton-mediated cell motility (27–38). One such enzyme is Rho-Kinase (ROK, also called Rho-binding kinase), which is a serine–threonine kinase (27–38). ROK interacts with RhoA in a GTP-dependent manner, and phosphorylates a number of cellular substrates including myosin light chain phosphatase, adducin, and LIM kinase (27–38). HA–CD44 interaction promotes RhoA-ROK activation in a number of tumor cells (27–38). Structurally, ROK is composed of a catalytic (CAT), a coiled-coil, a Rho-binding (RB), and a pleckstrin-homology (PH) domain (27–38). Overexpression of either the RB domain or the PH domains (dominant-negative forms) of ROK by transfecting cells with RB cDNA or PH cDNA blocks HA/CD44-specific phenotypic changes (27–38). Also, inhibition of RhoA-activated ROK by Y27632 treatment effectively blocks the HA/CD44-induced cellular signaling and functions (31). These findings suggest that selective activation of CD44 signaling (via small size- vs. large size-HA) induces different RhoA–ROK pathway-specific effects in normal keratinocytes and/or SCC.

#### Rac1-Activated PKNγ Signaling Events

**Figure 4:**
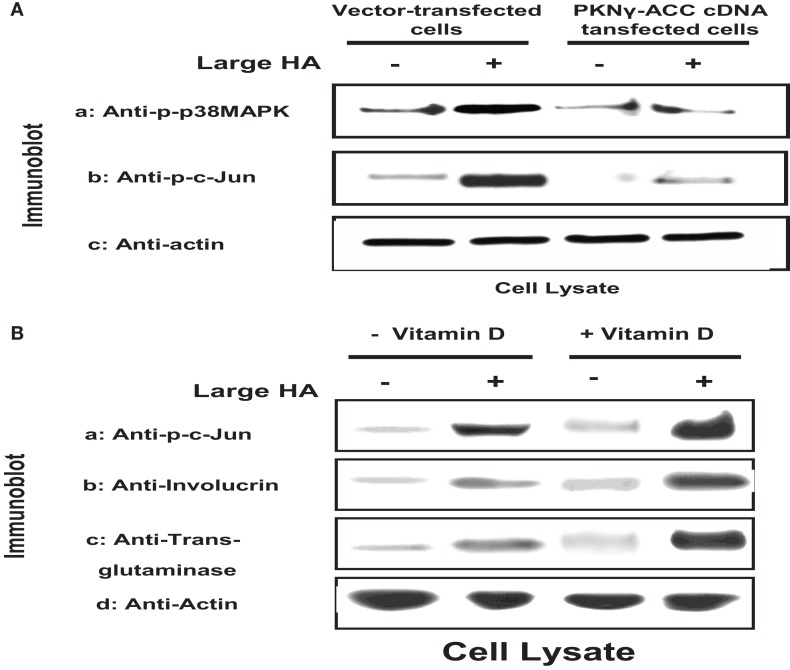
**(A)** Immunoblot analyses of large HA and PKN-dependent phosphorylation of p38MAPK and AP-1 proteins (e.g., c-Jun) [the PKN-dependent phosphorylation is proven by the effect of PKNγ-ACC cDNA dominant negative mutant], and **(B)** immunoblot analyses of large HA and vitamin D-induced c-Jun phosphorylation and differentiation marker (involucrin and transglutaminase) expression in cultured keratinocytes.

HA (mixed sizes) also promotes the interaction between CD44 and several Rac1-specific guanine nucleotide exchange factors [e.g., Tiam1 (32) and Vav2 (33)], thereby up-regulating Rac1 (another member of the Rho subclass of the Ras superfamily), leading to altered cytoskeleton-mediated cell functions (32, 33). Rac1 signaling has been shown to play an important role in promoting epidermal stem cells to undergo self-renewal and subsequent terminal differentiation (34). A number of enzymes have been identified as possible downstream effectors for Rac1 signaling. One such enzyme is protein kinase N-γ (PKNγ) (also called PRK2), which belongs to a family of serine–threonine kinases known to interact with Rac1 in a GTP-dependent manner. It also shares a great deal of sequence homology with protein kinase C in the C-terminal region (35–38). The N-terminal region of PKN contains three homologous sequences of approximately 70 aa (relatively rich in charged residues), which form an *a*ntiparallel *c*oiled-*c*oil fold (ACC domain) (35–38). This ACC domain has been shown to interact with RhoGTPases such as RhoA and Rac1 (and to a lesser extent with Cdc42) (35–38). The C-terminal region contains the C2-like region, which functions as an auto-inhibitory domain (35–38). Both the ACC and the C2-like domains, together with the catalytic domain, are conserved among the PKN family members (35–38). In keratinocytes, RhoA-activated PKNγ has been found to be involved in Fyn/Src kinase-regulated cell–cell adhesion during Ca^2+^-induced differentiation (39). Our results indicate that large HA specifically promotes CD44-mediated Rac1-PKNγ kinase signaling (3) and the activation of downstream effectors, including p38MAPK, AP-1 protein (c-Jun), and p63 (Figure 4). This pathway, in turn, regulates keratinocyte differentiation.

#### Hyaluronan, CD44, and RhoGTPases in Normal Keratinocytes and SCC

HA-mediated CD44 signaling has been shown to play an important role in various cellular functions. Our preliminary data indicate that small HA and large HA binding to cultured keratinocytes and SCC-12 cells selectively activates RhoA and Rac1 (Table 1). In particular, small HA (but not large HA) promotes RhoA signaling in cultured SCC-12 cells (to a lesser extent normal keratinocytes) (Table 1). Further analyses indicate that small HA-activated RhoA–ROK is capable of phosphorylating NFκB-p65 (Table 2), PKCε (Table 3), and Stat-3 (Table 3), leading to NFκB-specific (Table 2) and Stat-3-specific transcriptional activation (Table 3), cytokine (IL-6)/chemokine [monocyte-chemoattractant protein-1 (MCP-1)] gene expression (Table 2), and cell proliferation (Table 2). The fact that the ROK inhibitor, Y27632, significantly blocks small HA-induced RhoA–ROK signaling and SCC functions (Tables 2 and 3) suggests that small HA-mediated RhoA–ROK activation plays an important role in regulating oncogenic signaling events and SCC functions.

**Table 1:** **HA-induced RhoA and Rac1 activation in cultured keratinocytes and SCC12 cells**.

Samples	[^35^S]GTPγ•RhoA (cpm) (% of control)		[^35^S]GTPγ•Rac1 (cpm) (% of control)	
	Normal keratinocytes	SCC-12 cells	Normal keratinocytes	SCC-12 cells
No HA (control)	100 ± 2	100 ± 5	100 ± 3	100 ± 4
Small HA treatment	155 ± 5	**320 ± 10**	153 ± 2	127 ± 3
Large HA treatment	113 ± 3	110 ± 4	**313 ± 8**	129 ± 5

**Table 2:** **Small HA-mediated RhoA–ROK activation of NFκB-p65 and in cultured SCC-12 cells**.

Samples	NFκB-p65 phosphorylation (% of control)	NFκB-p65-specific-transcriptional activity (% of control)	IL-6/MCP-1 gene expression relative abundance (%)	Cell proliferation (% of control)
Untreated ROK (control)	100 ± 2	100 ± 2	0.74 ± 0.03/0.63 ± 0.01	100 ± 3
Large HA-treated RhoA-ROK	104 ± 3	106 ± 4	0.66 ± 0.02/0.69 ± 0.01	105 ± 5
Small HA-treated RhoA-ROK	**311 ± 14**	**315 ± 13**	**3.08 ± 0.10/2.88 ± 0.05**	**306 ± 10**
Small HA-treated RhoA-ROK + Y27632	153 ± 8	120 ± 3	0.60 ± 0.03/0.65 ± 0.04	127 ± 7
Small HA-treated RhoA-ROK + Vitamin D	140 ± 6	134 ± 5	0.63 ± 0.02/0.67 ± 0.03	105 ± 4

**Table 3:** **HA-mediated RhoA–ROK phosphorylation of PKCε and Stat-3 in cultured SCC-12 cells**.

Samples	PKCε phosphorylation (mol of Pi/mol of protein)	Stat-3-Ser (727) phosphorylation (% of control)	Stat-3-specific transcriptional activity (% of control)
Unactivated ROK	0.191 ± 0.02	100 ± 2	100 ± 3
Large HA-activated RhoA–ROK	0.193 ± 0.03	106 ± 4	104 ± 5
Small HA-activated RhoA–ROK	**1.254 **± 0.10	**265 ± 10**	**270 ± 10**
Small HA-activated RhoA–ROK + Y27632	0.136 ± 0.03	124 ± 7	136 ± 4
Small HA-treated RhoA–ROK + Vitamin D	0.158 ± 0.02	135 ± 8	128 ± 5

We also observed that the addition of large HA stimulates Rac1 activation in normal keratinocytes and to a lesser extent SCC (Table 1). In contrast, only a low level of Rac1 activation was detected in keratinocytes treated with small HA or no HA (Table 1). These findings suggest that large HA directly promotes Rac1 activation in normal keratinocytes. It is noted that the level of p38MAPK phosphorylation and AP-1 protein (c-Jun) phosphorylation is greatly enhanced in keratinocytes treated with large HA (Figure 4). Transfection of keratinocytes with PKNγ-ACCcDNA (but not vector-transfected keratinocytes) (Figure 4) or treatment of keratinocytes with Vitamin D not only inhibits large HA-mediated p38MAPK phosphorylation but also blocks AP-1 protein (c-Jun) phosphorylation in cultured keratinocytes (Figure 4). These results imply that the ACC fragment of PKNγ (similar as Vitamin D) acts as a dominant-negative mutant that downregulates large HA/CD44-induced Rac1-PKNγ activation and p38MAPK/c-Jun signaling required for normal keratinocyte functions. In addition, we have found that large HA promotes the expression of differentiation markers such as involucrin and transglutaminase in normal keratinocytes, and overexpression of PKNγ-ACC domain (or treatment of Vitamin D) also blocks differentiation marker expression as described previously (3). These findings suggest that large HA-mediated Rac1-PKNγ plays an important role in regulating p38MAPK-AP1 signaling and keratinocyte differentiation.

### Selective small HA-mediated CD44 isoform signaling that modulates acute/chronic UVR-induced inflammation in SCC, and determine the role of large HA in inhibiting small HA/CD44-mediated signaling and thereby preventing UVR-induced SCC progression

**Figure 5:**
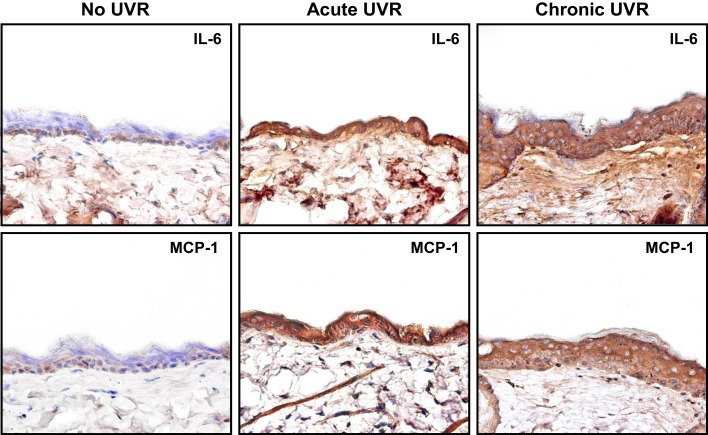
**Immunoperoxidase staining of cytokine (IL-6) and chemokine (MCP-1) in mouse skin following acute or chronic UVR exposure**.

There is compelling evidence that RhoA-activated ROK is involved in the regulation of oncogenesis (18). UVR has acute clinical effects on skin including inflammation. Pro-inflammatory cytokines and chemokines such as IL-6, IL-1β, RANTES, and MCP-1 contribute to inflammatory response in UVR-treated skin (40, 41). Our results indicate that upregulation of cytokine (IL-6) and chemokine (MCP-1) can be detected in mouse skin following acute or chronic UVR exposure (Figure 5). These findings clearly indicate a strong correlation between cytokine/chemokine expression and UVR-induced skin cancer.

The transcription factor, NFκB, is known to regulate genes involved in inflammatory responses (42). NFκB also controls cell cycle regulatory genes such as cyclin D1 and promotes cellular transformation (43, 44). Small HA (but not large HA)-mediated CD44 isoform interaction has been shown to play an important role in stimulating NFκB-inducing kinase (NIK). Activation of the inhibitor of κB (IκB) kinase (IKK) complex (IKKα and IKKβ) by NIK causes phosphorylation of the IκBs, targeting them for ubiquitylation and degradation by proteasomes, which liberate NFκB-p65 for nuclear translocation and transactivation of a variety of pro-inflammatory gene expression (21). Small HA also stimulates NFκB, which controls proinflammatory genes (e.g., IL-6, IL-1β, RANTES, or MCP-1 for inducing inflammatory responses) and cell cycle regulatory genes (e.g., cyclin D1 or c-Myc for activating cell proliferation) (42–44). A recent study indicates that RhoA signaling plays a role in activating NFκB signaling (45).

**Figure 6:**
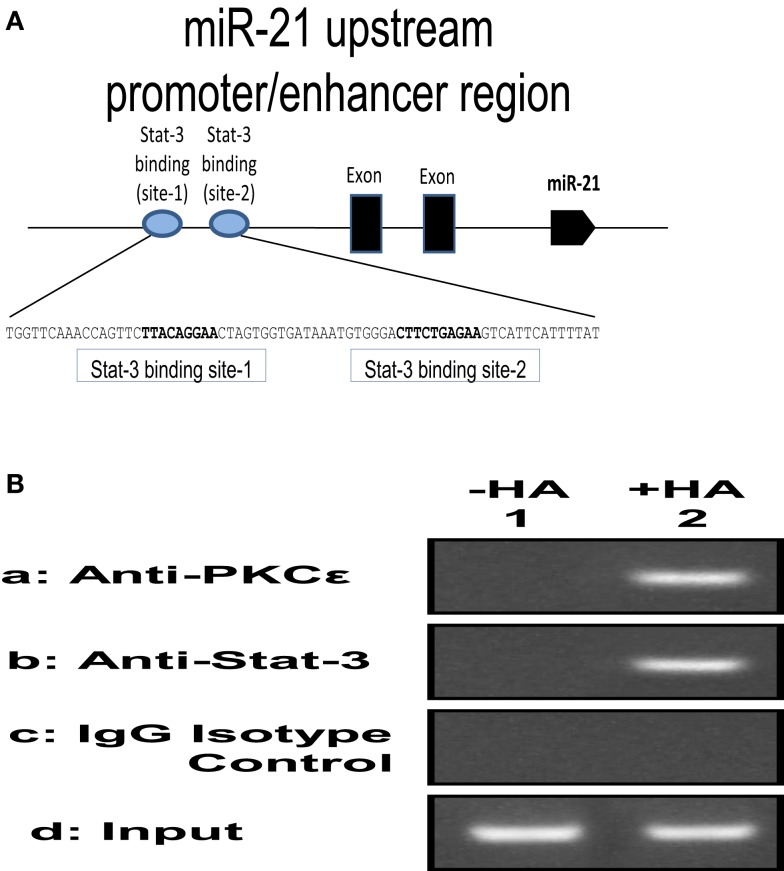
**(A)** Illustration of 130-bp regions containing two predicted Stat-3 binding sites (site 1 and site 2) upstream of the miR-21 genes; **(B)** ChIP assay of SCC-12 cells treated with no HA (lane 1) or with small HA (lane 2) using anti-PKCε, anti-Stat-3, or IgG control. Co-immunoprecipitated DNA was amplified by PCR with primers specific for the miR-21 upstream promoter enhancer.

UVR is also known to promote PKCε overexpression (46) and Stat-3 phosphorylation, leading to skin SCC development (47). Our preliminary data indicate that small HA promotes ROK phosphorylation of PKCε and Stat-3, which in turn stimulates Stat-3-specific transcriptional activities (Figure 6; Table 3) in SCC-12 cells. Large HA exerts direct anti-inflammatory and anti-proliferation effects on UVR-induced skin cancers. Whether large HA prevents small HA-mediated RhoA–ROK activation and NFκB-p65 signaling, as well as PKCε-Stat-3 function in transformed keratinocytes/SCC, and reverses small HA/CD44v-mediated tumor-specific behaviors (e.g., pro-inflammatory gene expression, cytokine/chemokine production, and proliferation) in transformed keratinocytes and SCC will be investigated in the future.

### MicroRNA-21 (miR-21) production and vitamin D 1,25(OH)_2_D_3_ effect on mouse skin following acute and/or chronic UVR exposure

Dysregulation of microRNAs (miRNA) is observed in many cancers including skin cancer. In particular, miR-21 appears to play an important role in tumor cell growth and SCC progression. Mature miRNA (e.g., miR-21) are detected in UVB-treated cells and skin carcinogenesis. In this study, we investigated the regulation and expression of miR-21 in mouse skin following acute and/or chronic UVR exposure as described below:

#### Identify HA/CD44-Mediated PKCε-Stat-3-Specific Target Genes

The mechanism of cellular transformation in keratinocytes and SCC is likely to be the results of several genes that are transcriptionally controlled by the PKCε-Stat-3 interaction. Previous studies indicate that activated Stat-3 up-regulates the mRNA levels of many genes, including miR-21. The expression of mature miR-21 is detected in various SCC cell lines and patient specimens. Many studies indicate that miR-21 may function as an oncogene and play a role in anti-apoptosis and tumorigenesis. A recent study indicates that the gene encoding oncogenic miR-21 is regulated by an upstream promoter/enhancer containing two Stat-3 binding sites (as diagramed in Figure 6). In order to investigate whether HA–CD44-induced PKCε-Stat-3 complex is involved in the regulation of miR-21 expression in normal/transformed human keratinocytes (SCC 12F2 and SCC12B2) or UVB-treated murine keratinocytes (from epidermal sheets), we conducted chromatin immunoprecipitation (ChIP) reporter gene assays as described below.

#### Chromatin Immunoprecipitation Assays

To examine whether the PKCε-Stat-3 complex directly interacts with the upstream promoter/enhancer region of miR-21, ChIP assays were performed in normal/transformed human keratinocytes (SCC12F2 and SCC12B2) or UVB-treated murine keratinocytes (from epidermal sheets) in the presence or absence of small/large HA (50 μg/ml) for 2 h at 37°C using a kit (EZ ChIP) from Millipore Corp., according to the manufacture’s instructions. Cross-linked chromatin lysates were sonicated and diluted with ChIP sonication buffer plus protease inhibitors, divided, and incubated with normal rabbit IgG or rabbit anti-PKCε antibody or rabbit anti-Stat-3 antibody at 4°C overnight, then precipitated with protein G-agarose. Cross-linking will be reversed by overnight 65°C incubation; DNA fragments then will be extracted with PCR purification kit, analyzed by PCR, and quantitated by real-time PCR using primer pair specific for the miR-21 upstream promoter/enhancer region containing the Stat-3 binding sites: forward primer: 5′-CTGGGAGAAACCAAGAGCTG-3′ and reverse primer:AGGGGACAAGTCAGAGAGAGG-3′ on an agarose gel as described previously (48). Results from these proposed experiments will allow us to verify the direct involvement of PKCε and/or Stat-3 in regulating the expression of specific target genes such as miR-21 in UVB-treated normal and transformed keratinocytes. Preliminary data indicate that PKCε and Stat-3 are recruited to the miR-21 upstream promoter enhancer region in SCC-12 cells treated with small HA (Figure 6).

#### Effect of MicroRNA-21 (miR-21) Production on Mouse Skin Following Acute and/or Chronic UVR Exposure

**Figure 7:**
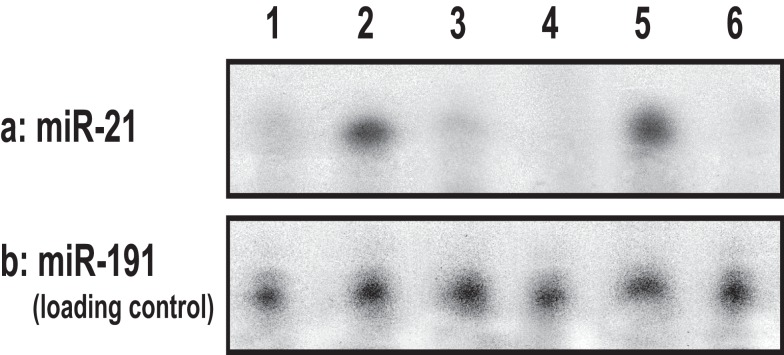
**UVB-induced miR-21 production using RNase protection assay in cultured keratinocytes [treated with no UVB (lane 1) or with UVB (lane 2) or treated with ROK inhibitor (Y27632) plus UVB (lane 3) or treated with UVB and vitamin D (lane 4)], and in UVB-induced SCC tumor tissues (lane 5) or normal skin tissues (lane 6)**.

Our results using RNase protection assays (Figure 7) show that the level of miR-21 production is significantly elevated in cultured keratinocytes treated with UVB (Figure 7, lane 2) as compared to those cells without UVB treatment (Figure 7, lane 1). The expression of Programed Cell Death 4 (PDCD4) gene is strongly induced during apoptosis in a number of cell types (49–51). PDCD4 encodes a tumor suppressor protein whose expression is lost in progressed carcinomas of many solid tumors (49–51). PDCD4 has been identified as one of the tumor suppressor genes regulated by miR-21 (48, 52, 53). It inhibits translation of PDCD4 by forming a complex with the translation initiation factor eIF4A (an RNA helicase) (54–56).

The “inhibitors of apoptosis family of proteins” (IAPs) constitute a family of at least nine proteins including survivin that block apoptosis by direct binding to caspases (57). Overexpression of IAPs (e.g., survivin) is thought to be linked to chemoresistance by suppressing apoptosis (57, 58). Our data indicate that HA–CD44-mediated Stat-3 activation induces up-regulation of survival proteins (surviving) expression leading to anti-apoptosis and multidrug resistance in epithelial tumor cells. The fact that downregulation of either PKCε or Stat-3 by treating SCC-12 cells with PKCε siRNA or Stat-3 siRNA blocks HA/CD44-mediated cyclin D1/survivin expression suggests that both PKCε and Stat-3 play important roles in the expression of all these proteins.

#### Vitamin D 1,25(OH)2D3 Effect on Mouse Skin Following Acute and/or Chronic UVR Exposure

Vitamin D 1,25(OH)_2_D_3_ and its analogs exert direct anti-inflammatory and anti-proliferation effects on UVR-induced skin cancers (59–62). A previous study indicated that one of the hormonally active vitamin D analogs (BXL-628) inhibits ROK membrane localization and activation in tumor cells (63). Recently, a study also indicates that vitamin D [via vitamin D receptor (VDR) action] was shown to abrogate the ability of NFκB-p65 to transactivate gene transcription and to block cytokine/chemokine gene expression as well as subsequent inflammatory responses and drug resistance in epithelial cells (64, 65). Our data indicate that 1,25(OH)_2_D_3_ (similar as large HA) (65) prevents small HA-mediated RhoA–ROK activation and NFκB-p65 signaling, as well as PKCε-Stat-3 function in transformed keratinocytes/SCC, and reverses small HA/CD44v-mediated tumor-specific behaviors (e.g., pro-inflammatory gene expression, cytokine/chemokine production, and proliferation) in transformed keratinocytes and SCC (Tables 2 and 3).

**Figure 8:**
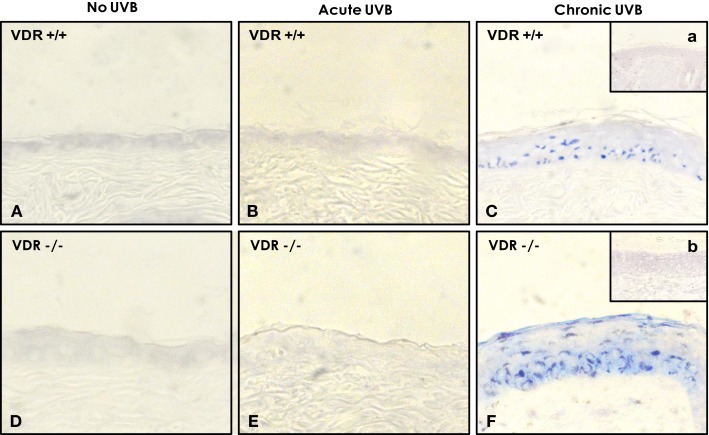
**Detection of UVB-induced miR-21 expression in wild-type (VDR^+/+^) and VDR-null (VDR^−/−^) mouse skin using DIG-labeled miR-21 probe (LNA probe) (A–F) (and scrambled probe) (a & b) and *in situ* hybridization [incubated with an anti-DIG-AP (alkaline phosphatase)-FAB fragment plus NBT/BCIP]**.

In addition, we found that cultured keratinocytes treated with either ROK inhibitor (Y27632) (Figure 7, lane 3) or vitamin D (Figure 7, lane 4) or normal skin tissues (Figure 7, lane 6) contain significantly less UVB-induced miR-21 [as compared with UVB-induced SCC tumor tissues (Figure 7, lane 5)]. The expression of miR-21 occurs in SCC tumor tissues (Figure 8) (but not in normal skin tissues) (Figure 8). Using digoxigenin (DIG)-labeled miR-21 probe (LNA probe) (and scrambled probe) and *in situ* hybridization [incubated with an anti-DIG-AP (alkaline phosphatase)-FAB fragment plus NBT/BCIP], we have detected an upregulation of miR-21 in VDR-null mouse skin (to a lesser extent wild-type mouse skin) following chronic UVR exposure. In contrast, very little miR-21 is detected in either VDR-null mice or wild-type mice following acute UVR or no UVR treatment (Figure 8). These findings suggest that VDR has a protective role to attenuate miR-21 production, and downregulation of VDR enhances miR-21 expression leading to tumor cell growth and skin SCC progression. These observations strongly suggest that UVB promotes miR-21 production in both cultured keratinocytes and SCC tumors. Our observations also support our hypothesis that vitamin D (together with ROK inhibition) may serve as a protective agent to impair UVB-induced miR-21 production and skin cancer progression.

### Determine how large HA/CD44 epican interactions with Rac1-activated PKNγ lead to modulations in epidermal functions, DNA repair, and keratinocyte survival in mouse epidermis following acute or chronic UVR-induced skin cancer development

A previous study showed that HA/CD44-mediated Rac1-PKNγ activation plays an important role in regulating keratinocyte differentiation (8). Our recent work indicates that large HA (but not small HA) stimulates keratinocye signaling (Figure 4). The question of how large HA/CD44-induced Rac1-PKNγ signaling pathway mediates keratinocyte differentiation is not well-understood. In mammalian cells, at least three distinct mitogen-activated kinases (MAPKs) [e.g., ERKs (extracellular signal regulated kinases), the stress-responsive JNK/SAPKs, and p38MAP kinases (p38MAPK)] have been characterized (66, 67). There is compelling evidence that activation of p38MAPK occurs in the membrane (68, 69). Activated p38MAPK has been shown to phosphorylate specific transcription factors and proteins including the transcription factor activator proteins (AP-1) (consisting of members of Jun family of nuclear proteins such as c-Jun, JunB, JunD, Fra-1, Fra-2, c-Fos, and FosB) (70–74). Subsequently, phosphorylated AP-1 proteins bind to DNA elements and induce target gene expression (72, 73). In keratinocytes, the promoter activity of several differentiation-related markers (e.g., involucrin and transglutaminase) and cholesterol synthesis-related proteins (e.g., HMG-CoA synthase and HMG-CoA reductase). Both cholesterol synthesis-related proteins (e.g., HMG-CoA synthase and HMG-CoA reductase) known to be involved in keratinocyte lipid synthesis and differentiation appear to be closely associated with p38MAPK and AP-1 phosphorylation (activated forms) (71–73). Our data indicate that large HA stimulates p38MAPK and c-Jun phosphorylation (Figure 4) as well as keratinocyte differentiation (Figure 4).

UV-induced DNA lesions are known to be repaired by nucleotide excision repair (NER) (74), which operates via either global genomic repair (GGR) or transcription-coupled repair (TCR) (75, 76). Loss of certain NER proteins such as xeroderma pigmentosum group C (XPC) results in a selective impairment of GGR (75, 76). The p38MAPK appears to be involved in activating p53 and p63 – both of which are key regulators of NER (75–80) and have been shown to play an important role in maintenance and induction of VDR and certain key DNA damage recognition proteins (e.g., XPC). Whether large HA/CD44-activated Rac1-PKNγ and p38MAPK are involved in regulating p63/p53 signaling leading to DNA repair and keratinocyte survival following UVR exposure is investigated in this study. Our results indicate that pretreatment of cultured keratinocytes with exogenously added large HA exhibited a 1.7-fold enhancement of cell survival (*P* < 0.0004) following UVB 708 (J/m^2^) treatment.

**Figure 9:**
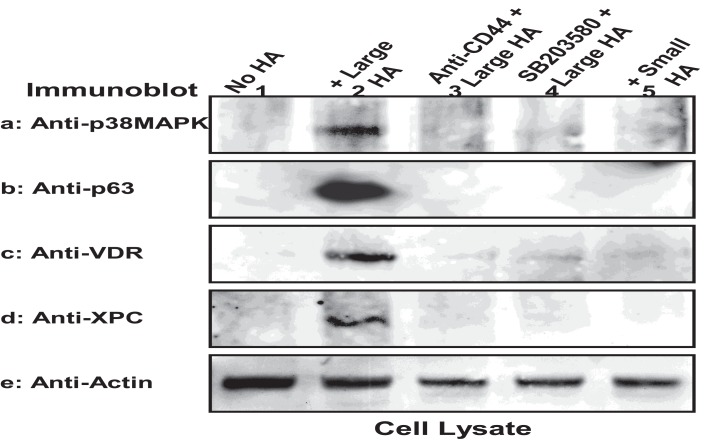
**Large HA-mediated upregulation of signaling regulators in keratinocytes**. Murine keratinocytes isolated from epidermal sheets were pretreated with HA (50 μg/ml) (lane 2) in the presence or absence of p38MAPK inhibitor (SB203580) for 24 h followed by irradiation with UVB (708 J/m^2^), harvested and analyzed by immunoblotting using anti-p38MAPK **(a)** or anti-p63 antibody **(b)** or anti-VDR antibody **(c)** or anti-XPC antibody **(d)** respectively. Actin was probed by anti-actin antibody as a loading control. (Cells were treated with no HA (lane 1) or with large HA (lane 20) or with anti-CD44 plus large HA (lane 3) or with SB203580 plus large HA (lane 4) or with small HA (lane 5) [p63, a p53-like transcription factor; VDR, vitamin D receptor; XPC, DNA repair protein].

These findings reveal selective activation of Rac1-PKN activation by large HA with pathway-specific effects on normal keratinocyte functions. In particular, we have discovered a novel signaling mechanism showing large HA stimulation of Rac1-PKNγ1 and phosphorylation of p38MAPK in cultured keratinocytes. It is possible that Rac1-PKNγ1 phosphorylated p38MAPK promotes AP-1 (c-Jun) (Figure 4) and p63/p53-mediated transcriptional upregulation (Figure 9), resulting in target gene expression and epidermal functions. We have found that the protein level of certain regulatory molecules such as p38MAPK, p63, VDR, and XPC is greatly enhanced in both cultured keratinocytes/epidermal sheets treated with large HA (but not small HA) (Figure 9). In contrast, the expression level of these key regulatory proteins (e.g., p38MAPK, p63, XPC, or VDR) is relatively low in keratinocytes treated with HA or p38MAPK inhibitor (anti-CD44 antibody and SB203580, respectively) plus large HA (Figure 9). These findings suggest that large HA/PKN-induced p38MAPK signaling plays an important role in both maintenance and induction of tumor suppressor proteins (e.g., p63) and VDR as well as certain key DNA damage recognition proteins (e.g., XPC). These findings support the notion that large HA/CD44 signaling events may interact with DNA repair pathways required for epidermal protection against UVB-induced skin damage.

**Figure 10:**
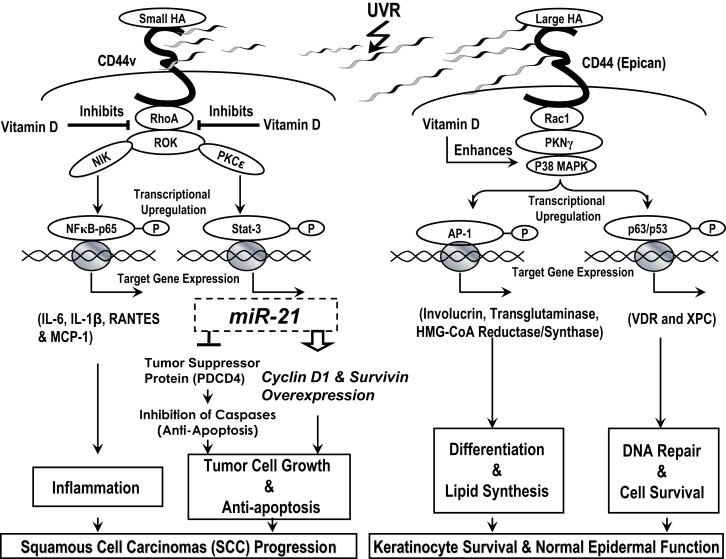
**A proposed model for UVR-induced hyaluronan (HA) degradation and selective HA (small HA vs**. large HA)-CD44 interaction with RhoA–ROK and Rac1-PKNγ in regulating normal and transformed keratinocyte signaling leading to healthy epidermal function and/or cutaneous squamous cell carcinomas (SCC) progression. Vitamin D enhances large HA-CD44-mediated differentiation, DNA r8epair, and keratinocyte survival/normal epidermal function, and it also inhibits small HA/CD44v-mediated RhoA–ROK signaling and SCC-specific tumor behaviors.

In summary, we would like to propose the following model (Figure 10): UVR induced HA degradation and selective HA (small HA vs. large HA)-CD44 interaction with RhoA–ROK and Rac1-PKNγ in regulating transformed and normal keratinocyte signaling leading to cutaneous SCC progression and/or healthy epidermal function. Vitamin D enhances large HA-CD44-mediated differentiation, DNA repair, and keratinocyte survival/normal epidermal function, and it also inhibits small HA/CD44v-mediated RhoA–ROK signaling and SCC-specific tumor behaviors.

## Summary and Outlook

To test our hypothesis, we provided new experimental evidence showing that administration of large HA or vitamin D-related treatments can selectively downregulate the CD44-RhoA/ROK-mediated inflammatory pathways and upregulate CD44-Rac/PKNγ-mediated cell differentiation, DNA repair, and keratinocyte survival function in mouse skin exposed to acute or chronic UVR. The ability for us to identify key HA (small vs. large HA)/CD44-mediated Rho/Rac signaling mechanism(s) by acute or chronic UVR in the regulation of keratincocyte functions will provide valuble insights regarding the possible HA/CD44 involvement of acute or chronic UVR-induced changes in influencing abnormal keratinocyte function, epidermal dysfunction, and tumor formation/SCC progression. In addition, we suggested that signaling perturbation agents (e.g., Y27623, a ROK inhibitor) can be applied to acute or chronic UVR-induced tumor formation/SCC progression displaying upregulation of keratinocyte inflammation and proliferation in order to correct the imbalance between RhoA–ROK signaling and Rac1-PKNγ activation during epidermal dysfunction.

In conclusion, we believe that the information obtained from these studies described in this article will allow us to delineate UVR-induced HA (small vs. large HA)-mediated CD44 signaling mechanisms and subsequent keratinocyte activities, as well as to provide important HA-based and vitamin D-related therapeutic approach regarding the treatment of UVR-induced keratinocyte transformation and skin cancer progression. The overall idea here is to develop innovative approaches to affect skin cancer growth and SCC progression that may be useful for future clinical studies. The new knowledge obtained from our HA/CD44-signaling strategies and HA-based/vitamin D therapeutic approaches should reveal new avenues for possible treatment of UVR-induced skin cancers.

## Conflict of Interest Statement

The authors declare that the research was conducted in the absence of any commercial or financial relationships that could be construed as a potential conflict of interest.
